# Deep learning in gonarthrosis classification: a comparative study of model architectures and single vs. multi-model methods

**DOI:** 10.3389/frai.2025.1413820

**Published:** 2025-02-05

**Authors:** Sahika Betul Yayli, Kutay Kılıç, Salih Beyaz

**Affiliations:** ^1^Artificial Intelligence and Digital Analytics Solutions, Turkcell Technology, Istanbul, Türkiye; ^2^Orthopedics and Traumatology Department, Adana Turgut Noyan Research and Training Centre, Baskent University, Adana, Türkiye

**Keywords:** artificial intelligence, deep learning, transfer learning, Kellgren–Lawrence, gonarthrosis, medical imaging, multimodal learning, CLAHE

## Abstract

**Purpose:**

This study aims to classify Kellgren–Lawrence (KL) osteoarthritis stages using knee anteroposterior X-ray images by comparing two deep learning (DL) methodologies: a traditional single-model approach and a proposed multi-model approach. We addressed three core research questions in this study: (1) How effective are single-model and multi-model deep learning approaches in classifying KL stages? (2) How do seven convolutional neural network (CNN) architectures perform across four distinct deep learning tasks? (3) What is the impact of CLAHE (Contrast Limited Adaptive Histogram Equalization) on classification performance?

**Approach:**

We created a dataset of 14,607 annotated knee AP X-rays from three hospitals. The knee joint region was isolated using a YOLOv5 object detection model. The multi-model approach utilized three DL models: one for osteophyte detection, another for joint space narrowing analysis, and a third to combine these outputs with demographic and image data for KL classification. The single-model approach directly classified KL stages as a benchmark. Seven CNN architectures (NfNet-F0/F1, EfficientNet-B0/B3, Inception-ResNet-v2, VGG16) were trained with and without CLAHE augmentation.

**Results:**

The single-model approach achieved an F1-score of 0.763 and accuracy of 0.767, outperforming the multi-model strategy, which scored 0.736 and 0.740. Different models performed best across tasks, underscoring the need for task-specific architecture selection. CLAHE negatively impacted most models, with only one showing a marginal improvement of 0.3%.

**Conclusion:**

The single-model approach was more effective for KL grading, surpassing metrics in existing literature. These findings emphasize the importance of task-specific architectures and preprocessing. Future studies should explore ensemble modeling, advanced augmentations, and clinical validation to enhance applicability.

## Background

Gonarthrosis, or knee joint degeneration, is a condition that causes movement restriction, stiffness, and pain, impacting quality of life, especially in older adults. It affects approximately 13% of women and 10% of men over the age of 60, and its incidence is increasing due to global aging trends, particularly in developed countries (Alkan et al., 2014; Shamekh et al., 2022). The key factors involved in its development include age, weight, comorbid diseases such as rheumatoid arthritis, trauma and genetic factors (Haberal et al., 2021; Osteoarthritis, 2023).

The diagnosis of gonarthrosis typically involves standing weight-bearing (WB) antero-posterior (AP) X-rays, which are considered the gold standard (Tiulpin et al., 2018). Magnetic resonance imaging (MRI) is more sensitive and specific than other methods, but X-rays remain a more economical and practical choice in clinical settings (Newman et al., 2022). Early treatment of gonarthrosis can prevent disease progression, but patients in advanced stages often require surgery. Gonarthrosis is recognized by the World Health Organization (WHO) for its role in increased mortality and rehabilitation necessity (Osteoarthritis, 2023).

The Kellgren–Lawrence (KL) system, the most prevalent radiological staging system for gonarthrosis, divides conditions into five stages (0–4) based on criteria such as joint space narrowing (JSN) and osteophyte formation. This staging system is somewhat subjective and semiquantitative (Kohn et al., 2016).

In light of the inherent subjectivity associated with the KL grading system, advancements in artificial intelligence present a considerable opportunity to mitigate these challenges. Advances in artificial intelligence have shown potential in improving the accuracy and standardization in medicine by leveraging extensive datasets to discern complex patterns, thus enhancing diagnostic precision and reducing human errors (Alowais et al., 2023).

### Related work and contributions

Numerous researchers have employed various artificial intelligence techniques to perform KL grading from AP knee radiographs. For instance, Olsson et al. developed a model using CNNs that takes images as input and outputs KL grades (Olsson et al., 2021). Similarly, Wang et al., Antony et al., and Chen et al. focused on cropping the knee joint area (region of interest) before training their models (Wang et al., 2021; Antony et al., 2017; Chen et al., 2019). Studies by Li et al. (2023) and Wang et al. (2021) have particularly highlighted the importance of preprocessing images by cropping the region of interest, showing that this step can significantly enhance model performance. Table 1 presents a comparison of studies employing direct CNN models for the detection and classification of knee osteoarthritis using the Kellgren–Lawrence system. It includes details about the datasets, imaging techniques, and specific CNN architectures used.

**Table 1:** Comparative analysis of studies with direct CNN approaches for KL classification.

Study	Approach	Dataset	Dataset size	X-ray position	Used architectures	ROI	F1 score	Accuracy	Precision	Recall
Our study	Direct CNN Approach	Self-created dataset, labeled by orthopedic surgeons	14,607	AP	NfNet F0, NfNet F1, EfficientNet B0, EfficientNet B3, Inception ResNet V2, VGG16	Yes	0.763	0.767	0.760	0.767
Antony et al. (2017)	Direct CNN Approach	OAI and MOST datasets	7,366	AP	Custom CNN network	Yes	0.610	0.603	0.610	0.630
Olsson et al. (2021)	Direct CNN Approach	Self-created dataset, labeled by orthopedic surgeons	6,103	AP, lateral, and oblique	ResNet	Yes	Not specified	Not specified	KL 0: 0.88 KL 1: 0.75 KL 2: 0.61 KL 3: 0.71 KL 4: 0.78	KL 0: 0.97 KL 1: 0.96 KL 2: 0.92 KL 3: 0.92 KL 4: 0.84
Chen et al. (2019)	Direct CNN Approach	OAI dataset	4,130	AP	ResNet, VGG, DenseNet, Inception	Yes	Not specified	VGG-19-Ordinal: Manually detected knee joints: 0.696 Automatically detected knee joints: 0.704	Not specified	Not specified

Building on these foundational techniques, subsequent research has delved into more complex methods of analyzing knee radiographs for osteoarthritis evaluation. The initial exploration of combining features from different regions to evaluate osteoarthritis was conducted by Tiulipin et al. They explored the use of a Siamese network to analyze features from both sides of the knee, processing each half independently with separate CNNs (Tiulpin et al., 2018). Lee et al. combined five different CNN models to enhance prediction accuracy (Lee and Kim, 2023), and Wei Li et al. input both AP and lateral knee radiographs into their CNN model for KL grading (Li et al., 2023). Khalid et al. enhanced feature extraction with CNNs by employing Principal Component Analysis (PCA) to eliminate unnecessary features before training a Feed Forward Neural Network (FFNN) to perform KL grading (Khalid et al., 2023). Yoon et al. went further by defining four regions of interest (ROIs) to assess osteoarthritis presence or absence and quantified joint space to classify osteophyte presence or absence, thus providing a comprehensive model to evaluate osteoarthritis and KL grades (Yoon et al., 2023). Table 2 outlines advanced approaches for knee osteoarthritis detection, featuring multi-model strategies, ensemble methods.

**Table 2:** Comparative analysis of studies with advanced approaches for KL classification.

Study	Approach	Dataset	Dataset size	X-ray position	Used architectures	ROI	F1 score	Accuracy	Precision	Recall
Our study	Multi-model strategy combining model outputs with demographic data and images	Self-created dataset, labeled by orthopedic surgeons	14,607	AP	NfNet F0, NfNet F1, EfficientNet B0, EfficientNet B3	Yes	0.736	0.767	0.735	0.740
Lee and Kim (2023)	Ensemble of 5 models	KneeXray dataset	8,260	AP	VGGNet, DenseNet, ResNet, TinyNet, EfficientNet, MobileNet, Xception, ViT	No	0.78	0.7705	0.79	0.71
Yoon et al. (2023)	Automated quantification of JSN and detection of osteophytes, KL grade classification	Osteoarthritis Initiative (OAI)	44,193	AP and Rosenberg	HRNet, RetinaNet, NASNet	Yes	–	0.830	KL 0 & 1: 1.00 KL 2: 0.63 KL 3: 0.77 KL 4: 0.89	KL 0 & 1: 0.80 KL 2: 0.91 KL 3: 0.74 KL 4: 0.94
Li et al. (2023)	Deep learning algorithm for detecting knee OA based on multi-view images and prior knee knowledge	Self-created dataset, labeled by radiologists	4,200	AP and lateral	U-Net, ResNet-50	Yes	0.970	0.970	0.970	0.970
Khalid et al. (2023)	Feature extraction with CNNs and PCA before KL grading with FFNN	OAI and Rani Channamma datasets	9,786	AP	FFNN, ResNet-101, VGG-19	Yes	0.99–0.98	0.98–0.98	–	–

Building on these advancements, our study primarily aims to enhance KL grading accuracy through a novel multi-model AI approach that incorporates both demographic data and clinical assessments. Our research is structured into two main subsections: the first applies a multi-model strategy to combine outputs from models analyzing osteophytes and JSN with demographic data and image; the second employs a traditional single-model approach for direct comparison. This structure will allow us to assess whether the multi-model approach with demographic information can outperform conventional methods in terms of diagnostic accuracy and reliability.

In training all models, image preprocessing involved cropping the region of interest, following the successful strategies of Li et al. and Wang et al. an object detection model was trained specifically to extract the ROI (Wang et al., 2021; Li et al., 2023).

Another significant contribution of our study is evaluating different model architectures to compare their performance for each specific task. We selected NfNet, EfficientNet, Inception-ResNet-v2, and VGG16 models for our transfer learning approach, chosen for their impressive performance in the ILSVRC competition and their distinctive architectural features (Brock et al., 2021). Details of these models are provided under the model selection section in the Materials and Methods.

Our third research question centers on comparing the performance of models trained with and without Contrast Limited Adaptive Histogram Equalization (CLAHE) in medical imaging. Building on the work of Pizer et al. (1990), who demonstrated how CLAHE enhances image clarity through localized histogram equalization in segmented regions using the Rayleigh distribution, and inspired by Mehdizadeh et al. (2023), who reported improvements in diagnostic accuracy for digital periapical radiographs, we explore the potential benefits of CLAHE for KL grading in osteoarthritis. Further influenced by Hayati et al. (2023), who observed variable effects of CLAHE across different model architectures in diabetic retinopathy classification, our study conducts experiments with two distinct augmentation sets—one incorporating CLAHE and one without. This approach allows us to systematically assess CLAHE’s influence on the performance of our models.

The objectives of our study can be summarized by the following research questions:

- RQ1: Does the proposed multi-model approach incorporating demographic inputs outperform the traditional single-model approach?
- RQ2: Which CNN model architecture yields the most successful results?
- RQ3: Does the application of CLAHE during image preprocessing enhance model performance?

## Methods and materials

### Study design

**Figure 1:**
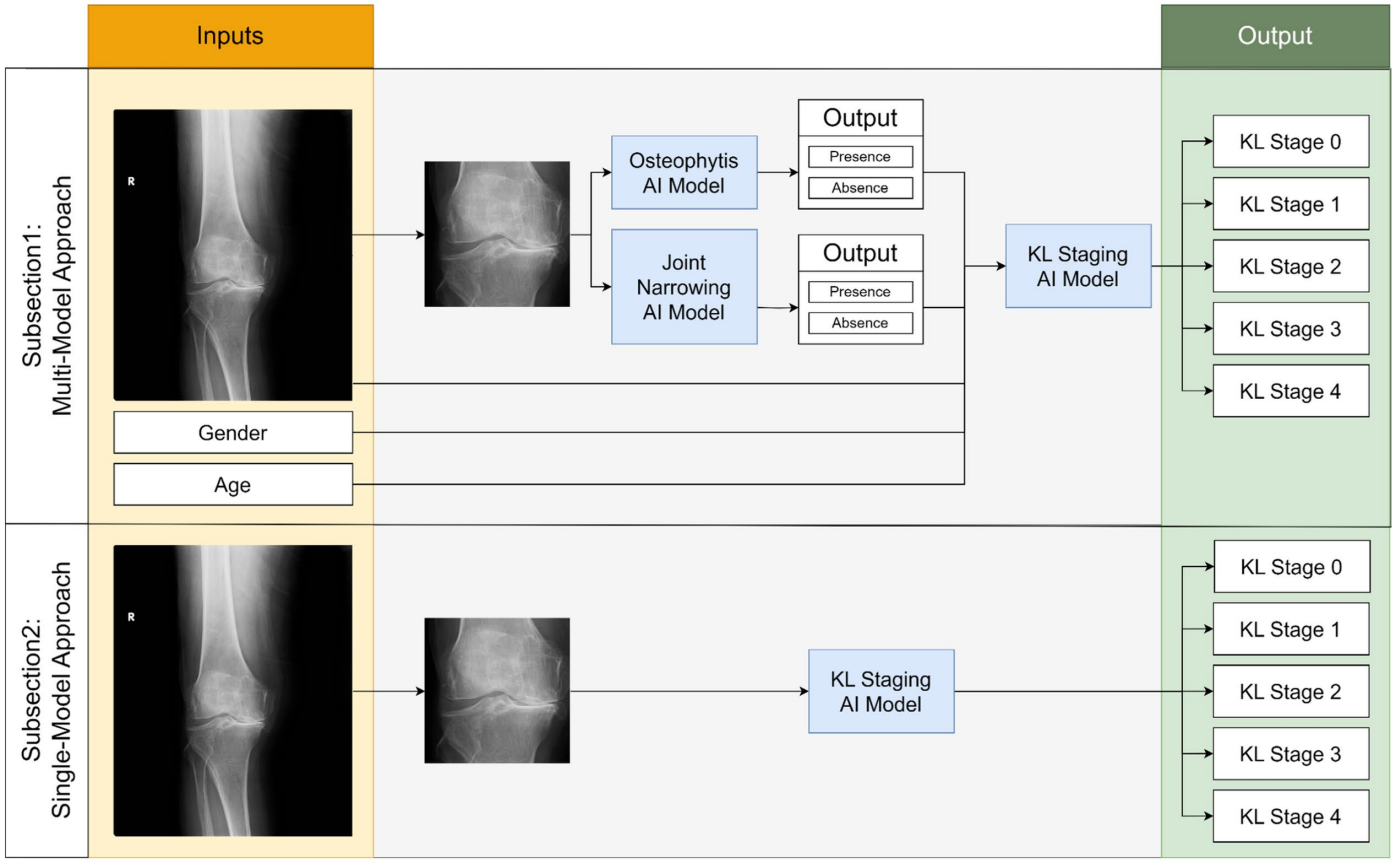
Simplified flowchart depicting two comparative experiments in our study for predicting KL stages of osteoarthritis.

Our study employed two main experimental approaches outlined in Figure 1:

1. Proposed multi-model approach: In this experiment, separate deep learning models were initially trained to identify specific pathological features, including joint narrowing and the presence of osteophytes. Subsequently, an integrated model was developed, combining these findings with the original knee X-ray images and demographic data (age and sex of the patient).
2. Single-model approach: The objective of this experiment was to evaluate the performance of deep learning models trained solely for direct KL grading from knee X-ray images. This approach served as a benchmark for comparison with the proposed multi-model approach.

To fulfill these objectives, our study included the training of deep learning models for four distinct cases as outlined above. Different model architectures were employed for each case to facilitate a comparative analysis of their performance. Additionally, to investigate the potential impact of the CLAHE method on image preprocessing, each model was trained using two different augmentation sets, one of which included CLAHE preprocessing.

In the Results section, we present the training outcomes for the four CNN models. Detailed evaluations of each model’s training process and performance metrics are provided in the subsequent subsections.

### Patient selection and data security

**Figure 2:**
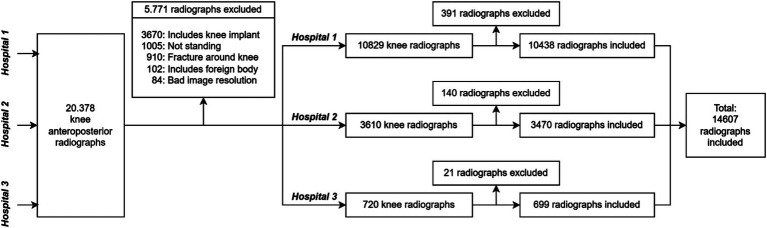
Flowchart detailing the dataset construction process for our study, including exclusion criteria and the number of radiographs considered at each hospital.

The database of Başkent University Hospital and its three affiliated hospitals were included in the study. A total of 20,378 knee X-ray images were taken between 2015 and 2021 from patients aged 18 and 100 years and were captured using seven different devices from four different manufacturers (Fuji, Kodak, Siemens, Philips). Images were excluded based on criteria such as presence of knee implants (3,670 images), not-standing position (1,005), fractures (910), foreign objects (102), poor quality (84), and unsuitability for KL grading (552), resulting in 14,607 images for analysis. Among these patients, 41% were male (average age 55.8 years), and 59% were female (average age 57.7 years). The selection of patients based on the centers and their demographic distribution is presented in Figure 2 and Table 3. All data were anonymized and stored securely. Transfers between institutions carried out using 256-bit encrypted hard drives to ensure privacy and compliance.

**Table 3:** Demographic distribution of patients from three different hospitals included in the study.

	Hospital 1	Hospital 2	Hospital 3	Total
Included images count	10.438	3.470	699	14.607
Men (%)	0.281	0.796	0.471	0.412
Men’s median age	53.132	59.261	51.006	55.827
Women (%)	0.719	0.204	0.530	0.588
Women’s median age	57.260	61.450	59.241	57.692

### Labeling of radiographs

The radiographs underwent labeling by three board-certified orthopedic surgeons, as detailed in a prior study. Two of these surgeons boasted more than a decade of experience, while the third had over 20 years of arthroplasty expertise.

We used the open-source CVAT software on our self-hosted servers to ensure secure and flexible data access (Sekachev et al., 2020). This choice enabled labelers to work seamlessly via a web browser, while also safeguarding data through the avoidance of external transfers.

**Figure 3:**
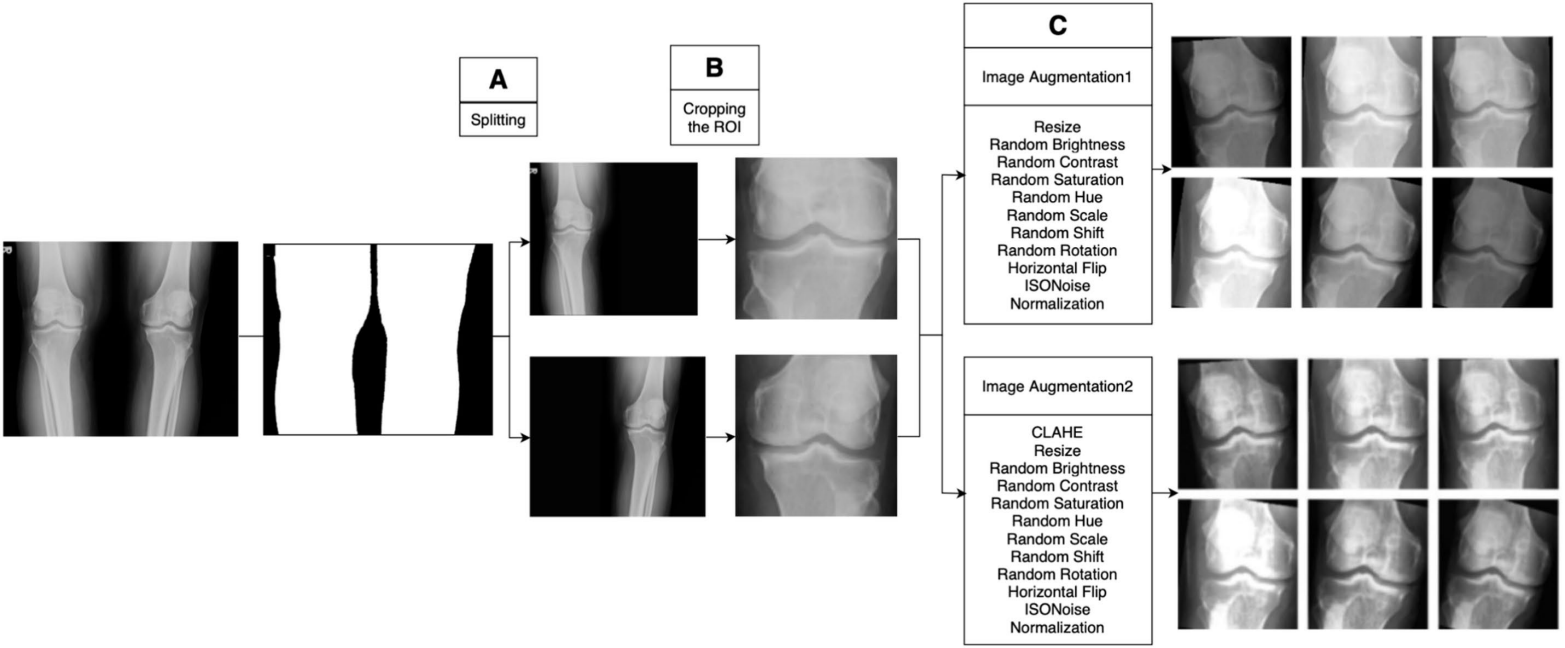
Image preprocessing stages. **(A)** Application of image processing methods for labeling, creating individual images for each extremity. **(B)** Use of an object detection model for precise cropping of the region of interest, ensuring consistency across all radiographs. **(C)** Examples of images post-augmentation.

We processed images of both extremities, separating them (as illustrated in Figure 3A) to ensure each knee could be individually annotated by the labelers. Each image was binarized by assigning a full pixel value to non-zero pixels. To remove artifacts, such as markers or noise, erosion and dilation processes were applied. After isolating the primary anatomical structure, the upper and lower midpoint coordinates were calculated and averaged to define a centerline. Using this centerline, each image was divided into two halves, with the opposite half blacked out in each image. This approach allowed clearer focus on each extremity and enhanced annotation accuracy.

The annotations included marking each radiograph for the KL gonarthrosis stage, presence of implants, osteophytes, and suitability for inclusion. Each labeler was capped at annotating 300 images per day to maintain precision. Through this process, a total of 552 radiographs were excluded based on predetermined criteria, as represented in Figure 2.

The final labels were determined as the average of assessments made by all three labelers, are concisely summarized in Table 4, showcasing the distribution of labels.

**Table 4:** Distribution of labels within the dataset used in the study.

	Hospital 1	Hospital 2	Hospital 3	Total
Included images count	10.438	3.470	699	14.607
KL Stage 0	242	8	8	258
KL Stage 1	3,188	1,325	283	4,807
KL Stage 2	3,442	275	264	5,031
KL Stage 3	2,163	1,336	107	2,796
KL Stage 4	1,403	526	37	1,715
Osteophyte +	4,610	1,064	206	5,883
Osteophyte −	5,828	2,406	493	8,724
Joint narrowing +	4,726	1,125	196	6,047
Joint narrowing −	5,712	2,345	503	8,560

### Image preprocessing

The image processing step is applied to enhance the performance of the deep learning model and achieve better results. We conducted image preprocessing in two steps before starting model training: cropping the joint areas in the radiographs and applying dynamic image augmentation techniques during model training (Figures 3B,C).

### Automatic detection of knee joint

We employed YOLOv5m model architecture, a member of the YOLO (You Only Look Once) family renowned for its rapid and accurate object detection capabilities (Jocher et al., 2022).

We created a mini dataset consisting of 500 randomly selected radiographs, adhering to radiologist standards for valid knee joint segmentation. This segmentation includes the area from the upper end of the tibia to the lower end of the femur, centering the cartilage in the image and potentially including parts of the fibula (Wang et al., 2021). The knee joint regions were annotated with bounding boxes. We divided the dataset into three parts: 300 for training, 100 for validation, and 100 for testing. We present model training results in Results section.

This custom-trained YOLOv5m model was subsequently applied to all the radiographs in our study, eliminating irrelevant anatomical structures and ensuring standardized image sizes throughout the dataset. The impact of this targeted cropping, transforming images from their pre-cropped to post-cropped state, is depicted in Figure 3B.

### Image augmentation

Image augmentation, a technique for diversifying the training dataset, enhances our model’s ability to generalize. This process is critical for reducing overfitting and improving model learning in a more generalized manner (Perez and Wang, 2017; Shorten and Khoshgoftaar, 2019).

Initially, we ensured that the input image sizes for each model matched those used during their original pretraining by applying a resizing process. Our study utilized two distinct augmentation sets, which were applied dynamically during the model training phase. A significant feature of our augmentation approach was the implementation of the contrast limited adaptive histogram equalization (CLAHE) technique. The parameters for our augmentation sets, including the application of CLAHE, are presented in Table 5, and examples of the augmented images are shown in Figure 3C.

**Table 5:** Parameters and their respective values for the data augmentation sets applied in our experiments.

	RandomBrightnessContrast		ShiftScaleRotate		ISONoise		HorizontalFlip		CLAHE		Normalize
	*p*	Limits	*p*	Limits	*p*	Limits	*p*	Limits	*p*	Limits	
Augmentation 1	0.5	Brightness limit: −0.3, 0.3 Contrast limit: −0.3, 0.3 Brightness by max: False	0.5	Shift: 0, 0.2 Scale: −0.2, 0.4 Rotate: −15, 15	0.3	Intensity: 0, 1 Color shift: 0.2, 0.5	0.5	–			Mean: (0.485, 0.456, 0.406) Std: (0.229, 0.224, 0.225)
Test Set Augmentation for Augmentation 1											Mean: (0.485, 0.456, 0.406) Std: (0.229, 0.224, 0.225)
Augmentation 2	0.5	Brightness limit: −0.3, 0.3 Contrast limit: −0.3, 0.3 Brightness by max: False	0.5	Shift: 0, 0.2 Scale: −0.2, 0.4 Rotate: −15, 15	0.3	Intensity: 0, 1 Color shift: 0.2, 0.5	0.5	–	1	Clip limit: 4,4 Tile grid size: 8,8	Mean: (0.485, 0.456, 0.406) Std: (0.229, 0.224, 0.225)
Test Set Augmentation for Augmentation 2									1	Clip limit: 4,4 Tile grid size: 8,8	Mean: (0.485, 0.456, 0.406) Std: (0.229, 0.224, 0.225)

### Data splitting

Our study employed two AI modeling methodologies, as detailed in Figure 1. To ensure robust and consistent evaluation across all tasks, a dedicated test set was separated at the beginning and held constant for use across the four tasks. This consistent test set provided a standardized basis for comparing model performances across different approaches.

The first methodology, a multi-model approach, consisted of two stages. In the first stage, separate models were trained to detect joint narrowing and the presence of osteophytes, each using 4,675 images for training and 1,168 for validation. In the second stage, a new AI model was trained to integrate the outputs of these initial models alongside the original images to produce final diagnostic outputs. This stage also used a distinct set of 4,675 training images and 1,168 validation images. Testing for all three cases was conducted using the initially separated test set, enhancing the robustness and reliability of the final model’s performance across tasks.

The second methodology, a single-step approach, used 9,350 images for training, 2,337 for validation, and tested on the same 2,920 images as the multi-model approach.

**Figure 4:**
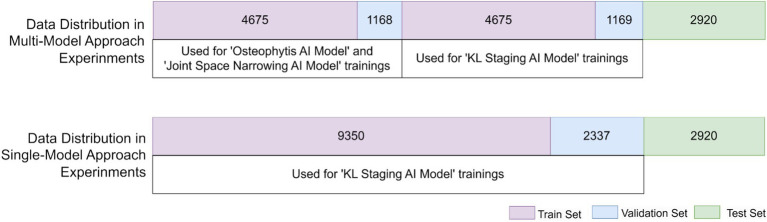
Distribution of dataset utilized in model trainings with two different approaches.

We also ensured proportional representation of KL grades across all datasets to maintain balanced training and testing, ensuring that each stage had the same distribution across all subsets, as illustrated in Figure 4.

### Model selection

In our study, we employed a transfer learning approach using models that have shown remarkable performance in the ImageNet Large Scale Visual Recognition Challenge (ILSVRC) (Brock et al., 2021). We selected the NfNet, EfficientNet, Inception-ResNet-v2, and VGG16 models, which are known for their distinctive architectural features and robust performance across ImageNet’s dataset, which includes approximately 1.3 million training images, 50,000 validation images, and 100,000 test images spread over 22,000 categories. This choice was motivated by their proven capabilities in handling diverse and complex image data, making them ideal for our objective of classifying KL stages in knee X-ray images.

- NfNet: Known for its high performance and architecture that omits normalization layers, NfNet utilizes adaptive gradient clipping for effective training without batch normalization (Brock et al., 2021). We trained NfNet-F0 and NfNet-F1 models.
- EfficientNet: Prioritizes computational efficiency and performance by introducing a scaling method for network dimensions and adopting the Swish activation function (Tan and Le, 2019). We trained EfficientNet-B0 and EfficientNet-B3 models.
- Inception-ResNet: This model combines the ability of the Inception architecture to capture complex patterns with ResNet’s residual connections, balancing performance and computational cost (Szegedy et al., 2016).
- VGG16: VGG16 features a deep structure effective for large-scale image classification and has demonstrated superior generalizability across various tasks and datasets (Simonyan and Zisserman, 2014).

### Training protocols

We utilized the PyTorch Image Models (timm) library, an open-source collection of state-of-the-art image models, pretrained weights, and utility scripts designed for training, inference, and validation in PyTorch (Wightman et al., 2023). Training was executed on NVIDIA Tesla V100 16 GB GPUs, which supported the computationally intensive demands of our protocols. Due to the variable distribution of data within our dataset, weighted sampling was implemented across all training iterations by enabling the relevant argument in the training function, allowing the model to give more focus to underrepresented data classes. This approach ensures that each class contributes proportionally to the learning process, thereby enhancing model effectiveness on less common data samples.

Each model was trained for 100 epochs using a learning rate of 10^−4^ and the Adam optimizer for efficient optimization. The models were stabilized using the weights from the best-performing epoch to ensure optimal performance.

### Statistical analysis

In our study, we assessed model performance using several key metrics.

Accuracy, defined as the proportion of true results (both true positives and true negatives) among the total number of cases examined, measured how well the model correctly identifies both positive and negative outcomes.

Precision (positive predictive value) and recall (sensitivity) evaluated the model’s ability to identify true positives and all relevant cases, respectively. We also calculated the weighted F1-score to balance precision and recall in our imbalanced datasets.

The area under the receiver operating characteristic (ROC) curve (AUC), ranging from 0.5 (no better than chance) to 1.0 (perfect discrimination), gauged the model’s discriminative ability across various KL grade stages.

The confusion matrix detailed the counts of true positives, false positives, and false negatives. These metrics collectively enhanced our understanding of the models’ diagnostic accuracy, crucial for the statistical analysis in our study.

The Kappa statistic, which ranges from −1 (no agreement) to 1 (perfect agreement), helped quantify the agreement between predicted and actual classifications; Kappa values are interpreted as follows: no agreement beyond chance (0.0), slight (0.01–0.20), fair (0.21–0.40), moderate (0.41–0.60), good (0.61–0.80), and excellent (0.81–1.0). A higher Kappa value suggests a high level of agreement with the ground truth, indicating the model’s reliability. This metric helps determine the practical applicability of our models in real-world settings, guiding improvements in model training and selection.

## Results

### Knee joint area detection

The model underwent training on the subset for 20 epochs within the PyTorch framework using transfer learning techniques. To maintain consistency across the dataset, images were resized to a uniform 640 × 640 pixels using the bicubic interpolation method provided by OpenCV’s Python module, cv2.

For the transfer learning phase, we initialized our model weights with those from models pretrained on the comprehensive COCO dataset. This approach allowed us to leverage the extensive variety of object recognition patterns present in COCO, enabling more robust feature detection in our radiographs.

To ensure high precision in our ROI detection, we established a confidence threshold of 0.80. Post-training, the YOLOv5m model demonstrated its ability to accurately identify the ROIs, achieving a mean Intersection over Union (mIoU) score of 0.92. This score reflects the model’s high reliability in detecting relevant anatomical features. Notably, the accuracy of detecting the knee region reached 100% in our test set.

We initially started our ROI detection experiments with YOLOv5 and, upon achieving the required performance, did not find it necessary to train newer versions of the YOLO family.

### Multi-model approach for KL stage prediction

#### Osteophytosis presence classification using an AI model

**Figure 5:**
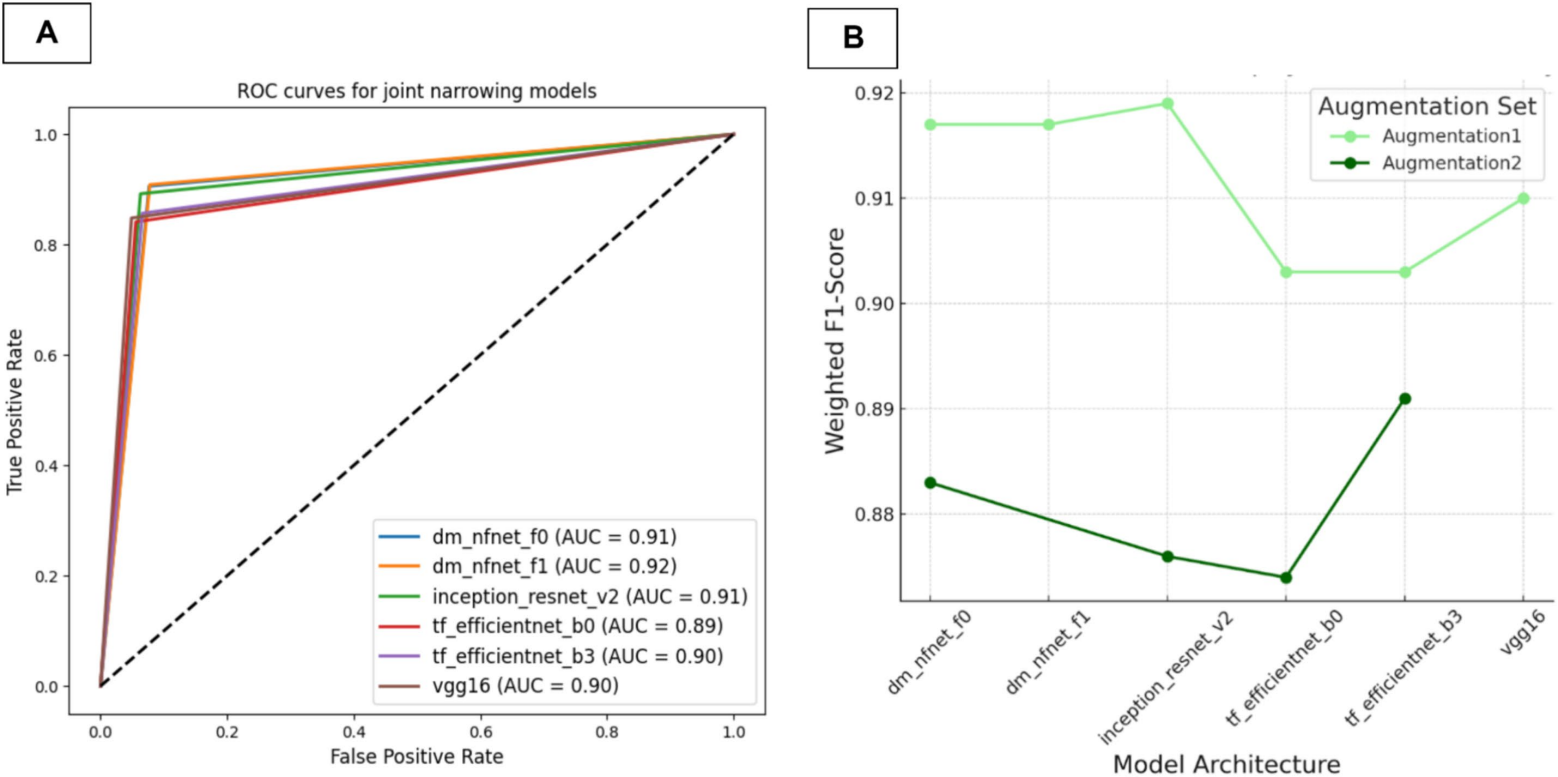
Performance evaluation of models for osteophyte classification. **(A)** Comparative ROC curves and AUC values. **(B)** F1-score plot for various models.

In this section, we evaluated the ability of various CNN architectures to detect osteophytes, training them with two distinct augmentation sets with the aim of identifying the most successful model for integration into a multi-model detection system. We’ve meticulously compiled the training parameters, image resolutions, and a full spectrum of performance metrics and Kappa coefficient, which are detailed in Table 6. The AUC values and ROC curves that provide insights into each model’s discrimination ability are presented in Figure 5A. The F1-scores of the trained models are presented in Figure 5B.

**Table 6:** Details of model configurations and evaluation results for osteophyte presence classification.

Model configuration details			Model evaluation metrics				
Model architecture	Augmentation set	Image size (pixels)	Accuracy	Precision	Recall	Weighted f1_score	Kappa coefficient
dm_nfnet_f0	Augmentation 1	192, 192	0.916	0.917	0.916	0.917	0.826
dm_nfnet_f0	Augmentation 2	192, 192	0.886	0.891	0.886	0.883	0.754
dm_nfnet_f1	Augmentation 1	224, 224	0.917	0.917	0.917	0.917	0.827
inception_resnet_v2	Augmentation 1	299, 299	0.919	0.919	0.919	0.919	0.831
inception_resnet_v2	Augmentation 2	299, 299	0.880	0.890	0.880	0.876	0.738
tf_efficientnet_b0	Augmentation 1	224, 224	0.903	0.904	0.903	0.903	0.796
tf_efficientnet_b0	Augmentation 2	224, 224	0.874	0.875	0.874	0.874	0.737
tf_efficientnet_b3	Augmentation 1	288, 288	0.903	0.903	0.903	0.903	0.797
tf_efficientnet_b3	Augmentation 2	288, 288	0.892	0.895	0.892	0.891	0.770
vgg16	Augmentation 1	224, 224	0.910	0.911	0.910	0.910	0.810

Results indicate that models trained without the CLAHE method (Augmentation1) consistently excelled across all architectures. According to Kappa and AUC metrics, all models demonstrated a strong agreement with ground truth.

**Figure 6:**
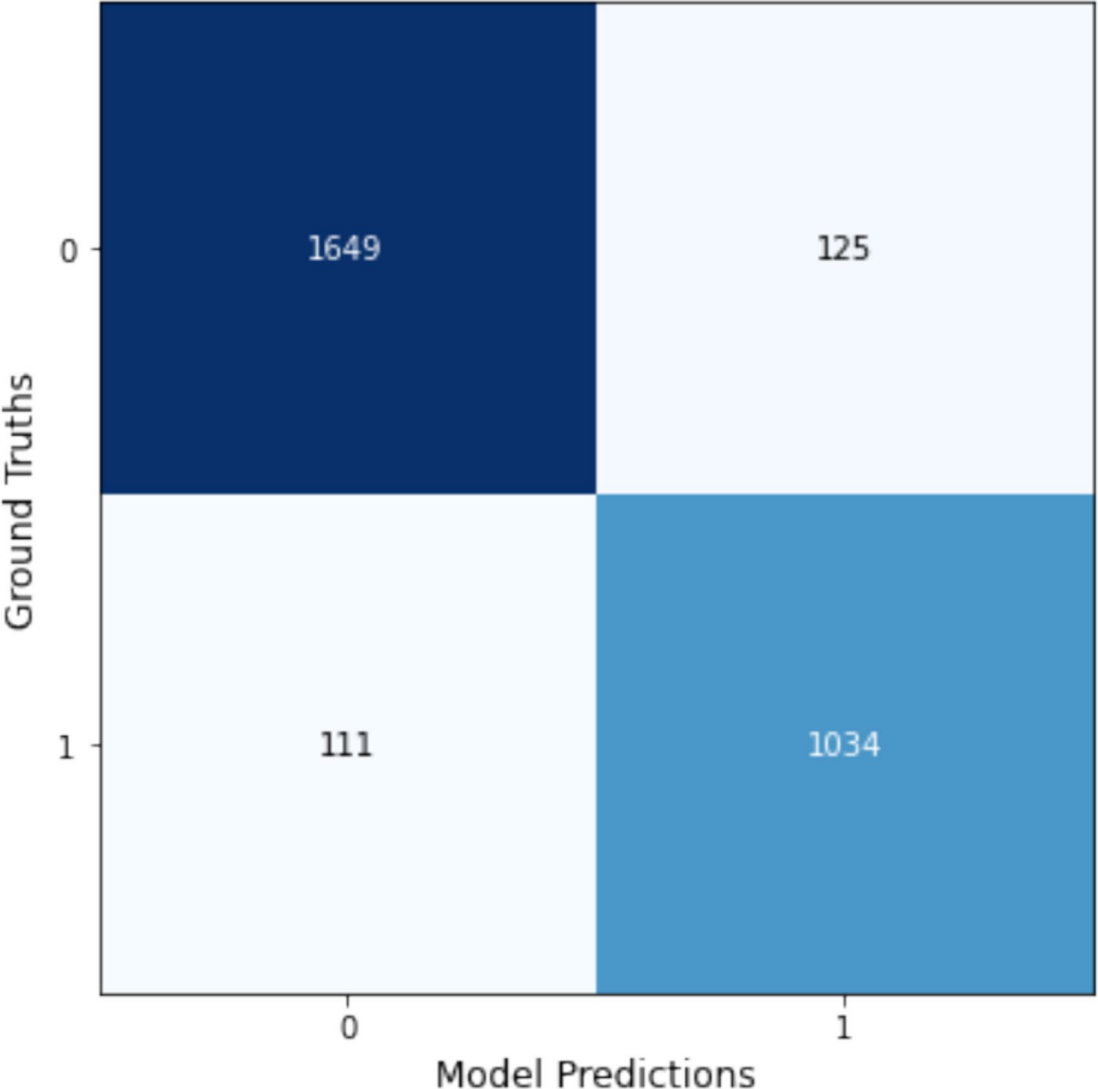
Confusion matrix for the Inception_ResNet_v2 model trained with Augmentation1, highlighting its effectiveness in osteophyte presence classification.

The Inception ResNet v2 model, which achieved the highest f1-score of 0.919 and Kappa score of 0.831, displayed superior consistency and reliability. Therefore, for its robustness and accuracy, this model was selected for integration into our multi-model framework. The corresponding confusion matrix for this model can be found in Figure 6, underscoring its statistically significant alignment with the ground truth.

#### Joint narrowing presence classification AI model

**Figure 7:**
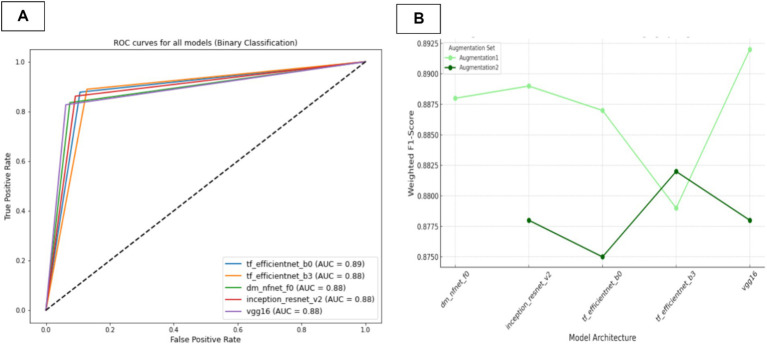
Performance metrics for models in JSN presence classification **(A)** Comparative ROC curves and AUC values. **(B)** F1-scores for the models.

In this section, we evaluated various CNN architectures for osteophyte detection. The models were trained using two distinct augmentation sets with the aim of identifying the most successful model for integration into a multi-model detection system. We meticulously compiled training parameters, image resolutions, and a comprehensive range of performance metrics, including the Kappa coefficient, as detailed in Table 7. Additionally, AUC values and ROC curves providing insights into each model’s discrimination ability are presented in Figure 7A. The F1-scores of the trained models are presented in Figure 7B.

**Table 7:** Details of model configurations and evaluation results for joint space narrowing presence classification.

Model configuration details			Model evaluation metrics				
Model architecture	Augmentation set	Image size	Accuracy	Precision	Recall	Weighted f1_score	Kappa coefficient
dm_nfnet_f0	Augmentation1	192, 192	0.889	0.888	0.889	0.888	0.767
inception_resnet_v2	Augmentation1	299, 299	0.889	0.889	0.889	0.889	0.770
inception_resnet_v2	Augmentation2	299, 299	0.879	0.880	0.879	0.878	0.746
tf_efficientnet_b0	Augmentation1	224, 224	0.887	0.888	0.887	0.887	0.767
tf_efficientnet_b0	Augmentation2	224, 224	0.877	0.879	0.877	0.875	0.739
tf_efficientnet_b3	Augmentation1	288, 288	0.879	0.882	0.879	0.879	0.752
tf_efficientnet_b3	Augmentation2	288, 288	0.883	0.884	0.883	0.882	0.754
vgg16	Augmentation1	224, 224	0.893	0.893	0.893	0.892	0.775
vgg16	Augmentation2	224, 224	0.880	0.884	0.880	0.878	0.745

The key finding was that models trained without the CLAHE method (Augmentation1) consistently outperformed those trained with it across all architectures, except for efficientnet-b3. According to Kappa and AUC metrics, all models demonstrated strong agreement with the ground truth.

**Figure 8:**
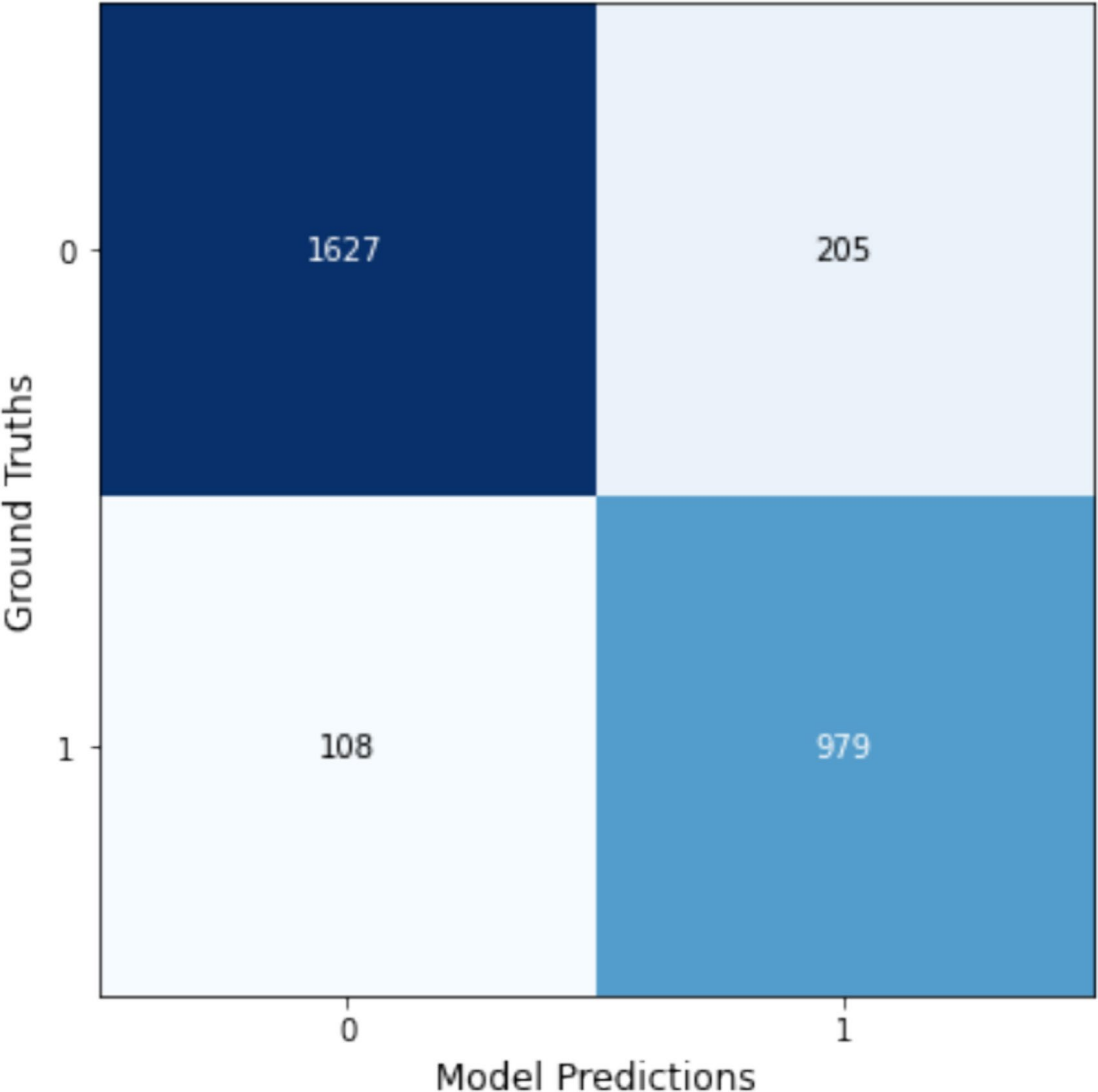
Confusion matrix for the VGG16 model using Augmentation1, showcasing its superior performance in classifying joint narrowing presence.

Based on these results, the VGG16 model trained with Augmentation1 achieved the highest F1-score of 0.892 and Kappa score of 0.775, indicating superior consistency and reliability. This model was deemed the most suitable for classifying JSN due to its high accuracy and robust performance. Consequently, it was selected for integration into our multi-model framework. The corresponding confusion matrix for this model can be found in Figure 8, further highlighting its statistically significant alignment with the ground truth.

#### Multi-input Kellgren–Lawrence grading AI model

In this analysis, we assessed the performance of a multi-input model designed to determine the KL stages. This model synthesizes various inputs including demographic information (age and sex), probabilities from osteophyte detection and joint space narrowing models, alongside X-ray images. We utilized an array of CNN architectures, each tested with two different data augmentation techniques, including one that incorporates CLAHE. Notably, certain models, including VGG16, Inception-ResNet-v2, and NFNet F1 under Augmentation 2, could not be implemented due to their computational complexity exceeding the capabilities of the available hardware. Consequently, we were unable to obtain and report the performance results for these models.

**Figure 9:**
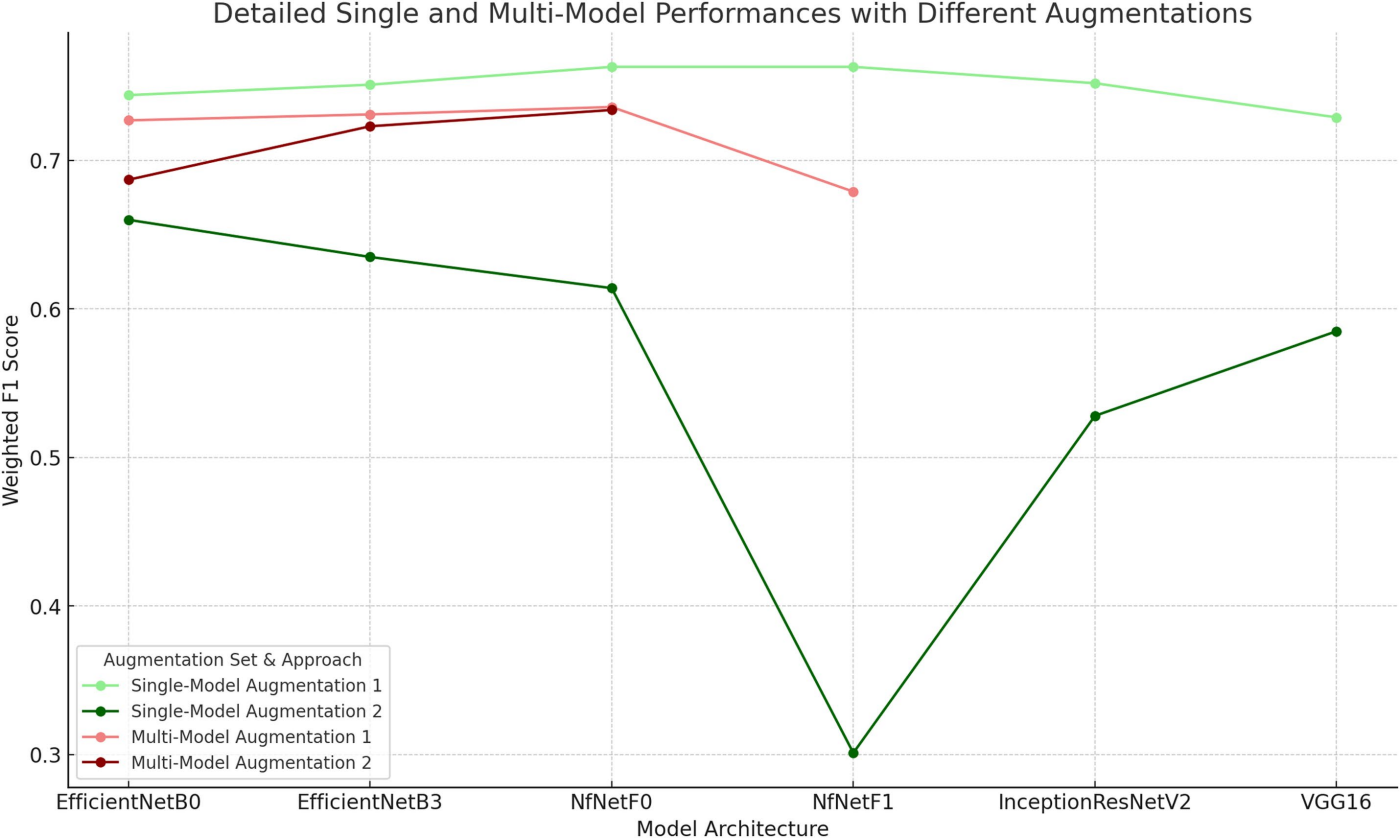
Weighted F1-Scores of Models with Different Architectures and Augmentation Techniques. The primary difference between Augmentation 1 and Augmentation 2 is the inclusion of CLAHE in the preprocessing pipeline for Augmentation 2.

**Figure 10:**
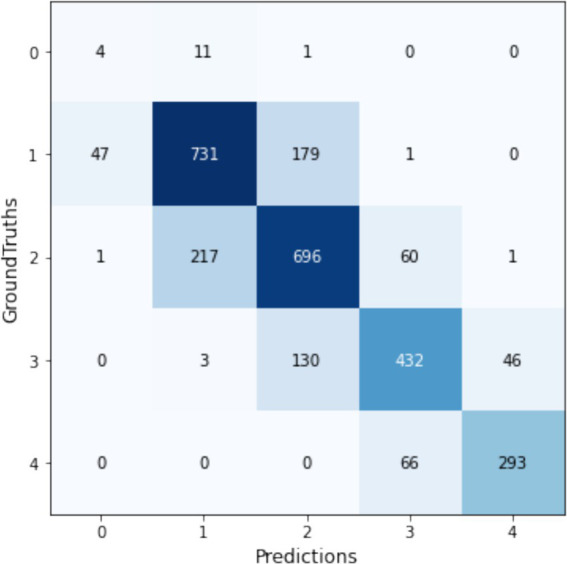
Confusion matrix of the NfNet_F0 model using Augmentation1, displaying its top performance in multi-input KL grading.

The performance of trained models was thoroughly assessed using multiple metrics, including accuracy, precision, recall, weighted F1 score, and Kappa value, detailed in Table 8. The F1-scores of the trained models are presented in Figure 9. Our analysis demonstrated that augmentation set without CLAHE achieved better results for all models, according to F1-scores and Kappa coefficients. The NfNet F0 model proved to be exceptionally effective, achieving the highest F1-score of 0.736 and a Kappa coefficient of 0.638 among the tested models when trained with Augmentation1. This F1-score indicates a commendable balance between precision and recall, signifying that the model is effective at correctly identifying true positives while minimizing false positives and negatives, which is crucial in medical diagnostics. The Kappa coefficient of 0.638 suggests a substantial agreement between the model’s predictions and the ground-truth labels, exceeding what would be expected by chance alone. The confusion matrix for this model is visually depicted in Figure 10.

**Table 8:** Details of model configurations and evaluation results for the multi-input model training in KL gonarthrosis grading, incorporating inputs of image, age, gender, osteophyte prediction, and joint space narrowing prediction.

Model configuration details			Model evaluation metrics				
Model architecture	Augmentation set	Image size (pixels)	Accuracy	Precision	Recall	Weighted f1_score	Kappa coefficient
dm_nfnet_f0	Augmentation1	192, 192	0.740	0.735	0.740	0.736	0.638
dm_nfnet_f0	Augmentation2	192, 192	0.739	0.732	0.739	0.734	0.636
dm_nfnet_f1	Augmentation1	224, 224	0.655	0.728	0.655	0.679	0.548
tf_efficientnet_b0	Augmentation1	224, 224	0.732	0.724	0.732	0.727	0.627
tf_efficientnet_b0	Augmentation2	224, 224	0.690	0.701	0.690	0.687	0.562
tf_efficientnet_b3	Augmentation1	288, 288	0.735	0.730	0.735	0.731	0.632
tf_efficientnet_b3	Augmentation2	288, 288	0.728	0.720	0.728	0.723	0.623

### Single-model approach for KL stage prediction

In the second phase of our investigation, we employed transfer learning techniques to train a series of deep learning models using knee X-rays, aiming to predict the Kellgren–Lawrence (KL) stages of gonarthrosis. The configurations of the models, including the specific augmentation strategies utilized and their corresponding performance metrics are detailed in Table 9. The F1-scores of the trained models are presented in Figure 9.

**Table 9:** Details of model configurations and evaluation results for training models in directly KL stage classification.

Model configuration details			Model evaluation metrics				
Model architecture	Augmentation set	Image size	Accuracy	Precision	Recall	Weighted f1_score	Kappa
dm_nfnet_f0	Augmentation1	192, 192	0.764	0.764	0.764	0.763	0.675
dm_nfnet_f0	Augmentation2	192, 192	0.616	0.665	0.616	0.614	0.475
dm_nfnet_f1	Augmentation1	224, 224	0.767	0.76	0.767	0.763	0.676
dm_nfnet_f1	Augmentation2	224, 224	0.286	0.544	0.286	0.301	0.218
inception_resnet_v2	Augmentation1	299, 299	0.756	0.75	0.756	0.752	0.662
inception_resnet_v2	Augmentation2	299, 299	0.52	0.621	0.52	0.528	0.368
tf_efficientnet_b0	Augmentation1	224, 224	0.744	0.746	0.744	0.744	0.646
tf_efficientnet_b0	Augmentation2	224, 224	0.649	0.688	0.649	0.66	0.531
tf_efficientnet_b3	Augmentation1	288, 288	0.755	0.751	0.755	0.751	0.661
tf_efficientnet_b3	Augmentation2	288, 288	0.63	0.69	0.63	0.635	0.5
vgg16	Augmentation1	224, 224	0.734	0.736	0.734	0.729	0.633
vgg16	Augmentation2	224, 224	0.578	0.638	0.578	0.585	0.439

**Figure 11:**
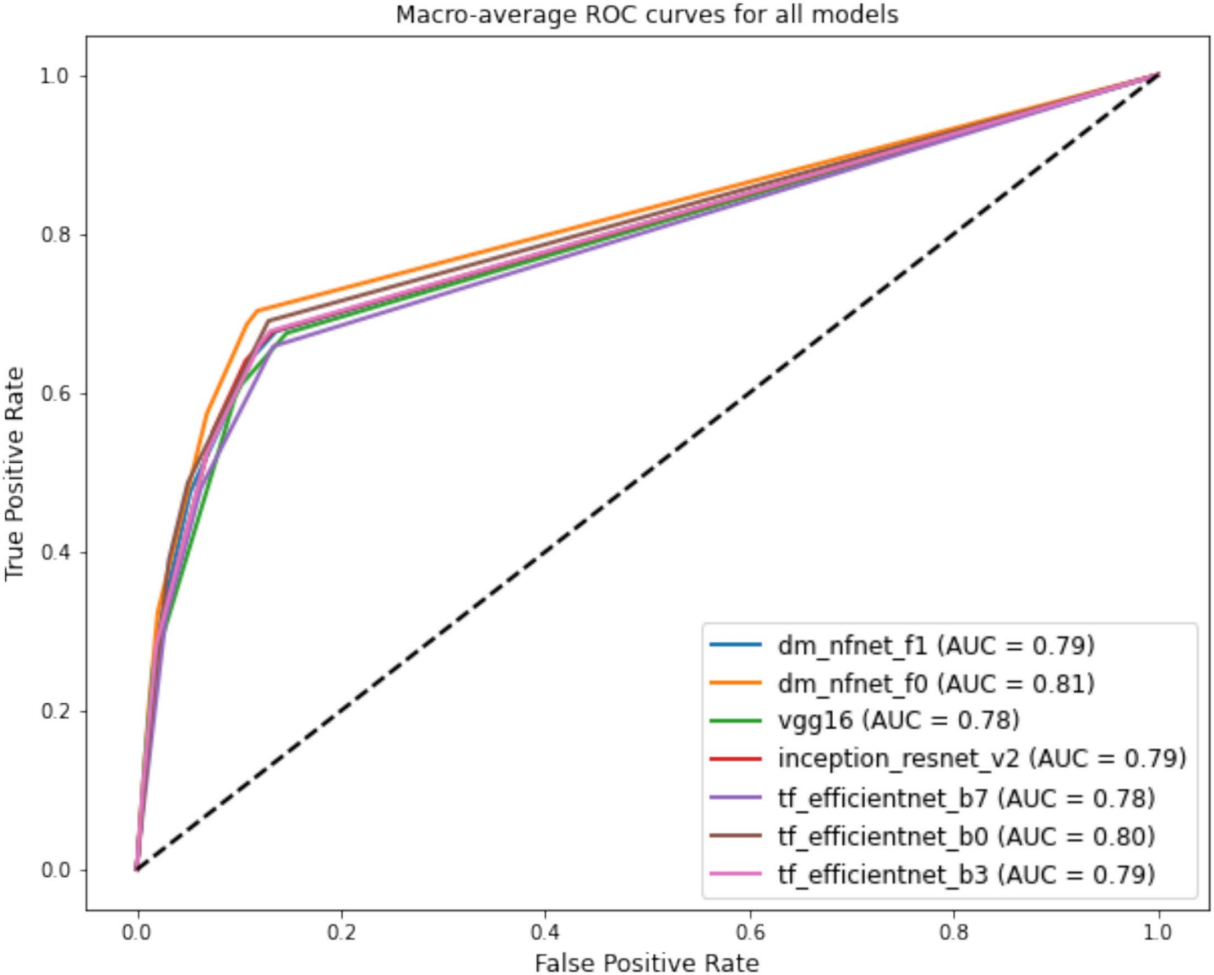
Comparative weighted ROC curves for deep learning model architectures trained with Augmentation Set 1, used in KL gonarthrosis staging.

Our findings indicate that models trained with the first set of augmentations, which excluded CLAHE (referred to as Augmentation1), consistently surpassed those trained with the second augmentation set (Augmentation2). The predictive performance of these models is further illustrated through the macro-average AUC and ROC curves presented in Figure 11. Specifically, the models utilizing Augmentation1 demonstrated high consistency in performance, as reflected by closely aligned F1-scores and Kappa coefficients.

**Figure 12:**
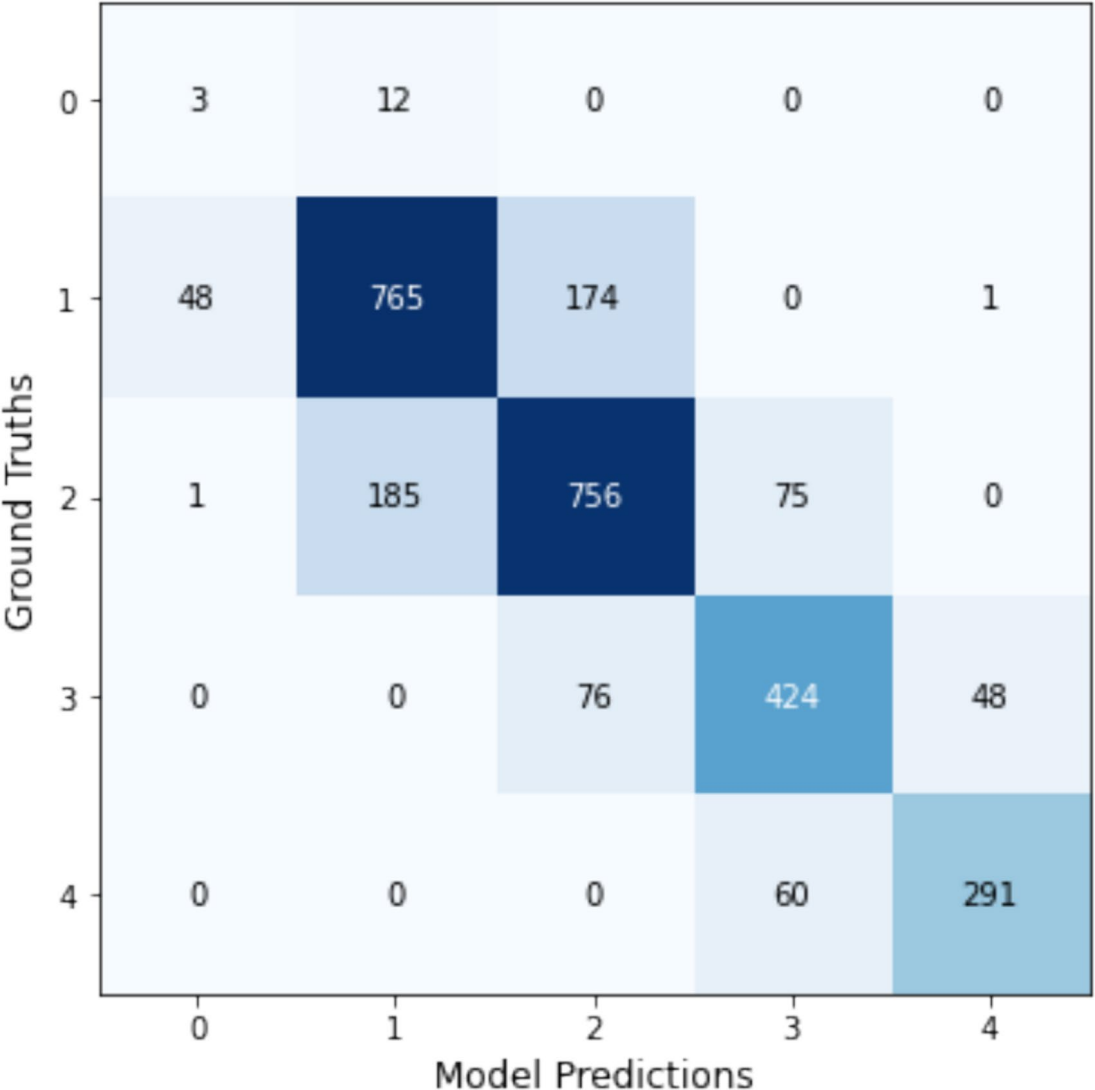
Confusion matrix for the dm_nfnet_f1 model trained with Augmentation1, showcasing its top performance in KL gonarthrosis staging.

Notably, the ‘dm_nfnet_f1’ model from the NfNet series, trained using Augmentation1, achieved the highest overall accuracy and F1-score, at 76.7 and 76.3%, respectively. Further insights into this model’s classification capabilities are illustrated through a confusion matrix in Figure 12. In contrast, employing CLAHE in the training process (Augmentation2) with the same CNN architecture resulted in a substantial decline in model performance, with the F1-score reducing dramatically to 30.1%.

Figure 10 presents a comparative graph of the weighted F1-scores for CNN models trained using both single-model and multi-model approaches to stage KL progression. The graph illustrates the performance metrics of models trained with two distinct augmentation sets: Augmentation1 and Augmentation2. It’s evident from the graph that single-model approaches with Augmentation1 (represented by the light-green line) maintain relatively stable F1-scores across different models. Conversely, the performance declines markedly for the single-model approach with Augmentation2 (dark-green line), particularly for the ‘dm_nfnet_f1’ model. In contrast, multi-model approaches exhibit a consistent performance irrespective of the augmentation set used (indicated by red lines), sustaining higher weighted F1-scores than the single-model with Augmentation2. The error bars represent the variability in the F1-scores, indicating the precision of the model performance estimates.

## Discussion

In our study, we assessed two distinct AI approaches for KL grading: a single-model method using only X-ray images and a multi-model strategy that integrates osteophyte detection, joint space narrowing (JSN) assessment, and demographic data. The single-model approach demonstrated superior performance across various architectures, achieving a significant 2.7% higher F1-score compared to the multi-model strategy in the best-performing models. This suggests that the simpler single-model approach may be more effective in extracting and leveraging the features relevant to KL grading, as shown in Figure 9, where models trained without CLAHE performed best.

Furthermore, models directly performing five-level classification (single-model approach) were observed to be more successful. This could be due to the fact that contemporary CNN architectures are already proficient in feature extraction, and adding demographic data along with specific information from additional models on osteophytes and joint space narrowing may have introduced bias into the multi-models.

According to our findings, all models trained with the single-model approach outperformed those from similar studies in the existing literature. This superior performance could potentially be attributed to the unique characteristics of our self-created dataset or the optimality of our model parameters. Our dataset, meticulously labeled by expert orthopedic surgeons, might possess qualities that particularly align well with the features our models are conditioned to recognize.

Furthermore, the best-performing models in single and multi-model approaches achieved kappa scores above 0.6, indicating a substantial agreement with the ground truth. This highlights that both methods, despite their performance differences, are reliable and could be clinically viable.

In our architecture-specific findings, NfNet consistently emerged as the most effective, performing well in both single and multi-input model configurations. Inception ResNet v2 and VGG16 excelled in the tasks of osteophyte presence and joint narrowing prediction, respectively. The close performance of other models suggests that multiple architectures have the potential to achieve high accuracy in these tasks, depending on the specific setup and implementation.

A pivotal aspect of our research was the exploration of different data augmentation sets while keeping other training parameters constant. Contrary to several previous studies advocating the benefits of CLAHE for enhancing model performance, our results consistently showed that models trained without CLAHE outperformed those that included it. This might indicate that CLAHE, by overly processing images, could disrupt the natural learning processes of sophisticated models, leading to overfitting on non-representative image features.

The significant decrease in the F1 score observed in the CNN model trained using the single-model approach with the NfNet f1 architecture may be attributed to overfitting, potentially induced by the application of CLAHE. Unlike the NfNet F0 model, theNfNet NfNet F1’s increased complexity and higher number of parameters might make it more susceptible to overfitting. This aspect highlights the need for careful consideration of model complexity when applying image enhancement techniques like CLAHE. Further research should explore this phenomenon in detail to better understand the trade-offs between model complexity and generalization capabilities in medical imaging tasks.

To summarize our key findings:

- We introduced a custom YOLOv5m detection model tailored for high-accuracy knee joint detection.
- Our self-created dataset allowed our single-model NfNet F0 to surpass performance metrics reported in existing literature.
- The single-model approach consistently outperformed our proposed multi-model strategy.
- NfNet F0 generally showed the highest success in our tests, while EfficientNet B0 often displayed lower performance.
- Except in one case, the application of CLAHE degraded the performance across all models.

These outcomes suggest that in the development of AI models for medical diagnostics, the choice and configuration of data preprocessing methods are as critical as the selection of the model architecture itself. This underscores the importance of a tailored approach to both data handling and model training to optimize diagnostic accuracy and model reliability.

While our multi-model framework was conceptually aimed at harnessing multiple sources of information—such as osteophyte detection, joint space narrowing outcomes, and demographic factors—our findings indicate that this approach did not surpass the simpler single-model strategy. One likely explanation is the challenge of data fusion: the sub-model outputs and demographic data may not have been optimally weighted or integrated, leading to partial redundancy or incomplete synergy in the final prediction. This can manifest as overfitting, where the model relies on spurious patterns stemming from sub-model inaccuracies rather than genuinely complementary features. For instance, our observation that including osteophyte and JSN predictions did not enhance KL staging suggests that these auxiliary signals introduced excessive complexity or noise.

Future research could improve multi-model approaches by employing attention mechanisms, learned gating networks, or joint training protocols that explicitly align sub-model features. Likewise, a deeper investigation into how demographic and radiographic features interact at different fusion layers could mitigate overfitting and reveal more meaningful synergies. Ultimately, refining these fusion strategies may help bridge the gap between multi-model comprehensiveness and the robust simplicity of a single end-to-end CNN.

## The role of bias, ethics, and multimodal clinical data in AI-based osteoarthritis classification

One of the biggest challenges in classification studies based on semi-objective criteria, such as Kellgren–Lawrence gonarthrosis grading, is achieving the most accurate labeling of the data. Inter- and intra-observer reliability has always been a matter of debate, and the impartiality of the labelers directly affects the accuracy of the results. In our study, the data were labeled by three experienced orthopedic surgeons, who routinely evaluate radiographs in daily practice and have direct exposure to degenerative findings during surgery. To minimize bias, a consensus approach was employed, with disagreements resolved by majority voting. Furthermore, the dataset includes images from multiple imaging devices and demographic groups, reducing potential biases related to imaging variability or population diversity. While orthopedic surgeons may be thought to favor surgical intervention, studies on interobserver reliability suggest that their evaluations remain consistent and reliable, mitigating such concerns.

In our study, data obtained from three different hospitals and eight different X-ray devices over an eight-year period were used to ensure impartiality and increase dataset diversity. This approach aims to enhance the generalizability of our findings and their relevance to real-life clinical scenarios. While the development of AI algorithms as a decision support system for radiologists is expected to reduce workload, it may also influence radiologists’ objective decision-making, potentially leading to ethical concerns. It is important to note that while radiological classification is a critical factor in treatment decisions, it is not the sole determinant. Incorporating additional clinical data, such as the patient’s age, weight, and treatment expectations, alongside radiological findings, would contribute to more balanced and impartial decision-making processes.

## Limitations

1. Model parameter optimization: To optimize each model for the task, it would have been ideal to experiment with multiple parameter configurations and select the most effective combination. However, due to time constraints, we were unable to conduct extensive hyperparameter tuning for each model.
2. Hardware limitations in multi-model approach: The models in the multi-model approach required more complex architectures. While we were able to train models with fewer parameters, our hardware could not support training models with a larger number of parameters, limiting the comparison of models we could experiment with.
3. Specificity to KL staging: Our study focused solely on the staging system, not incorporating other relevant staging systems that might provide a broader understanding of gonarthrosis.
4. Black-box nature of CNN: The CNN models used classify images without elucidating the specific features or locations related to osteophyte presence and JSN, limiting the depth of analysis compared to segmentation-focused techniques.
5. Objective measurements: We did not quantify the medial or lateral joint space areas, relying instead on clinically evaluated radiographs. This approach might affect the precision of our joint space assessments due to the lack of standardized radiography.
6. Radiographic scope: Only AP views were included, omitting the comprehensive three-dimensional aspects of the knee structure. As a result, conditions like lateral and patello-femoral joint arthrosis could not be assessed.
7. Clinical validation: The algorithms were not compared directly with clinical assessments by doctors, focusing instead on model development. Clinical validation against professional medical evaluations is planned for future research.
8. Exclusion criteria: Radiographs showing implants near the joint area were excluded, potentially limiting the application of our findings to the broader spectrum of patients, particularly those with posttraumatic arthrosis or implant.

## Future directions

Future studies could consider ensemble modeling, which combines outputs from multiple models to increase stability and accuracy. Additionally, implementing automated segmentation techniques for more refined ROI extraction may enhance predictive accuracy, especially in complex cases with overlapping features.

Further research could explore advanced augmentation techniques beyond CLAHE, such as elastic deformations to mimic anatomical variations or random rotations to account for minor positional shifts in X-rays. These methods may help improve model robustness by introducing naturalistic variability without altering core image features.

## Conclusion

In summary, our study introduces a pioneering approach to knee joint assessment through a two-tiered AI model using X-ray imagery, deep learning, and patient data. Initially, our models efficiently predicted KL stage using osteophyte formation and JSN indices. However, the multi-model strategy did not outperform the simpler single-model approach, indicating the need for further optimization.

Unique to our research is the development of a diverse custom dataset, collated from various hospitals and X-ray machines, offering a broad and unbiased perspective. This approach enhances the study’s robustness and sets a new standard in dataset creation for orthopedic AI research.

However, the reliance of the study on the subjective KL grading system introduces potential biases, emphasizing the need for ongoing research and validation with more diverse datasets to confirm the effectiveness and applicability of our models in real-world scenarios.

Overall, while our study marks progress in AI for orthopedic assessments, continuous research and validation are vital to refine these methods for practical clinical use, contributing to advancements in patient care in orthopedics.

## Data Availability

The raw data supporting the conclusions of this article will be made available by the authors without undue reservation.
